# The development of a glossary of contemporary Chinese values and its initial application

**DOI:** 10.3389/fpsyg.2023.1208366

**Published:** 2023-07-31

**Authors:** Li Pan, Xiting Huang

**Affiliations:** ^1^Faculty of Psychology, Southwest University, Chongqing, China; ^2^Research Center for Psychology and Social Development, Southwest University, Chongqing, China

**Keywords:** values, culture, multidimensional scale, Chinese, internalization

## Abstract

A value is an outward or inward view of what is “worthwhile” and is a belief system that people uses to distinguish between good and bad and to guide their behavior. Values vary from culture to culture and from time to time. In order to develop a vocabulary for measuring the values of contemporary Chinese, an open-ended survey was conducted in Study 1 to collect the value vocabulary of the Chinese public, and a text analysis was conducted in Study 2 to acquire the value vocabulary of the Chinese society. In Study 3, a word list of contemporary Chinese values was developed by integrating the words obtained from the above two studies and combining words from previous studies, and a preliminary application of the word list was conducted. The results revealed that Chinese values include four dimensions: *self-fulfillment*, *self-cultivation*, *social development,* and *interpersonal ethics*. Chinese values are characterized by diversity, and some of the socially advocated values have been internalized into the value system of individuals.

## 1. Introduction

Personal values are broad desirable goals that motivate people’s actions and serve as guiding principles in their lives. More importantly, they influence people’s perception, cognition, and behavior over time and across situations (Sagiv and Schwartz, 2022). Personal values have been explored in depth by numerous researchers. Researchers have developed cross-culturally adapted theories of values and have developed widely adapted values measurement instruments. For instance, Rokeach (1973) categorized values into two dimensions, terminal values and instrumental values, and developed the Rokeach Values Survey (RVS). Schwartz (1992) focused more on the motivational characteristics of values and improved on Rokeach’s categorization of values. Ten basic values (*self-direction, stimulation, hedonism, achievement, power, security, conformity, tradition, benevolence, universalism*) and four higher-order values (*openness to change, self-enhancement, conservation, self-transcendence*) were then proposed, as well as the development of the Schwartz Values Survey (SVS). Subsequently, Schwartz et al. (2012) refined the values theory by expanding the original 10 basic values to 19 values and developed the Portrait Values Questionnaire (PVQ). Moreover, after analyzing the data from the World Values Survey, Inglehart (2006) divided the values of human society into two dimensions, namely, traditional values-secular-rational values, and survival values-self-expression values. Several researchers have applied Western value theories and value scales to explore the structure and characteristics of Chinese values. For instance, Huang et al. (2012) utilized the RVS to examine the characteristics of the values of young students in five cities in China. Meanwhile, Lian and Guan (2014) investigated the values of deaf college students in China using the SVS. In another instance, Yang et al. (2019) adopted PVQ to explore the value characteristics of ethnic minorities in southwest China. The aforementioned value questionnaires have strong cultural adaptability and can reflect the characteristics of Chinese values. Nevertheless, values have cultural differences, and people living in different cultures exhibit different values. Moreover, the same values can mean different things in different cultures. For example, Schwartz and Cieciuch (2022) described that self-enhancement values have different meanings in different cultures. In addition, earlier studies have identified that the structure of Chinese values was not completely consistent with Schwartz’s theoretical structure of values (Jin et al., 2019). Therefore, the exploration of Chinese values should not be completely dependent on Western value measurement tools.

Thus, researchers in different regions of China have conducted empirical studies on the localization of Chinese values. In Hong Kong, China, The Chinese Culture Connection (1987) compiled the Chinese Values Survey (CVS). They further postulated that Chinese values have four dimensions: Integration, Confucian work dynamism, Human-heartedness, and Moderation. In the Taiwan Province of China, Yang (1994) compiled the Multidimensional Scale of Chinese Individual Traditionality (MSCIT) and the Multidimensional Scale of Chinese Individual Modernity (MS-CIM) and established that Chinese people displayed both traditional and modern values. In mainland China, Jin et al. (2009) developed the Chinese Values Questionnaire (CVQ) and determined that Chinese values are characterized by good personal orientation. Indeed, these value questionnaires do measure Chinese values. It is worth noting that over time, a number of modern values have emerged in the Chinese value system (Weng et al., 2021). In particular, China has been vigorously promoting the core socialist values since 2012, which reflect the “greatest common denominator” by the people of all ethnic groups in China (Xi, 2018). Additionally, numerous studies have evinced that contemporary Chinese people positively identify with core socialist values (Yu and Wang, 2013; Gao, 2016; Zhao and Wang, 2019), which means that some contents of the core socialist values may have been internalized into the personal value system. However, the content of socialist core values is less addressed in the existing value scales.

In summary, the content of Chinese values may differ from that of Western values due to cultural differences. And the established Chinese values measurement instruments were less likely to include the socially advocated values of contemporary Chinese. Therefore, it is imperative to develop a value-measuring tool that not only conforms to the characteristics of the Chinese nation but also to the characteristics of contemporary Chinese individuals.

The psycho-lexical approach assumes that language can express values (Sagiv and Schwartz, 2022). Currently, a number of researchers have used this method to explore the structure and characteristics of values in different cultural contexts and have confirmed the validity of the method (Borg et al., 2016; De Raad et al., 2016). Therefore, in this study, we used the psycho-lexical approach to collect contemporary Chinese values vocabulary in an attempt to examine the structure and characteristics of contemporary Chinese values from a bottom-up perspective. In Study 1, we conducted an open-ended survey to collect 56 words that reflect the values of contemporary Chinese public. In Study 2, we undertook a text analysis of *Xi Jinping: the governance of China*, from which we obtained 56 words that reflect the values advocated by contemporary Chinese society. In Study 3, we integrated the words obtained in Studies 1 and 2 and the values words from previous studies to form a list of 76 contemporary Chinese values words, and used the list to explore the characteristics of contemporary Chinese values initially.

## 2. Study 1: an open-ended survey of Chinese values

### 2.1. Materials and methods

#### 2.1.1. Participants

A total of 1,100 Chinese participants were enrolled in this survey, including 952 valid data. Among them, there were 486 males and 466 females, with an age distribution ranging from 15 to 67 years (*M* = 22.33, *SD* = 4.41). The participants came from all walks of life, comprising doctors, teachers, engineers, students, waiters, farmers, and so on. In terms of educational attainment, secondary education or below, tertiary education, and master’s degree and above accounted for 14.92%, 73.00%, and 12.08% of all participants, respectively. Participants in the survey came from 27 provinces on the Chinese mainland.

#### 2.1.2. Procedure

The study was conducted on the online platform of questionnaire star (China’s questionnaire collection website). During the survey, the researchers published a recruitment poster online every day, and the participants responded to the questions by clicking the questionnaire link or scanning a QR code. The question was, “What is most important to you personally?” Without specific restrictions on the answers, the participants could write one value word or multiple value words. Each participant was given 2 RMB as compensation.

### 2.2. Results and discussion

Frequency statistics were carried out on the collected words in the open survey. Firstly, the BICOMB 2.0 software (a Chinese word frequency statistics software) was employed for preliminary statistics of frequency. Then, words with similar meanings were combined and summarized. When multiple words expressed similar meanings, the word with the highest frequency was retained. Finally, 56 value words were obtained, with a total frequency of 1,603 (Table 1).

**Table 1:** Words obtained by open-ended survey and their frequency.

Values	Frequency	Values	Frequency
亲情(family affection)	332	地位(status)	8
健康(health)	268	机遇(opportunity)	8
金钱(money)	99	忠诚(loyalty)	8
快乐(happiness)	98	奉献(contribution)	7
友情(friendship)	95	和谐(harmony)	7
爱情(romantic love)	77	负责(responsibility)	6
自由(freedom)	70	人脉(well-connected)	6
诚信(honesty)	41	信任(trust)	6
能力(capacity)	40	毅力(willpower)	6
爱国(patriotic)	38	聪明(intelligent)	5
事业(enterprise)	33	法治(rule of law)	5
理想(ideal)	28	剬正(justice)	5
乐观(optimism)	23	平等(equality)	5
自信(self-confidence)	23	权力(power)	4
进取(aggressive)	22	稳定(stable)	4
独立(independent)	21	享乐(hedonist)	4
安全(safety)	19	容貌(appearance)	3
勤奋(diligent)	19	坦荡(frank)	3
渊博(erudite)	19	助人(mutual help)	3
信仰(belief)	18	自强(self-reliance)	3
真诚(sincerity)	16	创新(innovate)	2
陪伴(accompany)	15	富强(prosperity)	2
尊重(respect)	15	环保(environmental)	2
友善(kind-hearted)	14	开放(openness)	2
尊严(dignity)	13	踏实(earnest)	2
自爱(self-love)	10	报恩(gratitude)	1
勇敢(brave)	9	沟通(communication)	1
自律(self-discipline)	9	敬业(dedication)	1

As listed in Table 1, the top five items that individuals valued most were *family affection, health, money, happiness, and friendship*. Among them, *family affection* ranked first, which was in line with traditional Chinese familism (Yang, 2006). It was also consistent with the results of previous research on Chinese values in the sense that Chinese people attach importance to family (Li et al., 2018). *Health* also played a vital role for Chinese people. Various studies have established that individuals attach great importance to health (Wang and Sun, 2017; Wang T. et al., 2022). On the other hand, public health emergencies can increase individuals’ attention to health (Wei et al., 2020). This study was conducted in September 2021, when the COVID-19 epidemic was not over; hence, participants paid considerable attention to health. *Happiness* reflected the pursuit of individual spirituality, whereas *friendship* and *family affection* reflected the needs of contemporary Chinese people in terms of affection; *money* reflected the pursuit of material things. Taken together, these values mirrored the characteristics of the Chinese people’s pursuit of physical and mental integration as well as materialistic and spiritual life in this era. Several researchers have debated that contemporary Chinese people have formed a bicultural self, i.e., the individual-oriented self and the social-oriented self (Lu and Yang, 2006; Yang, 2006). Thus, Chinese values have both individual and social orientations (Jin et al., 2009). As anticipated, the value words reported by the participants in this study consisted of both individual orientation value words, such as *money* and *happiness,* and social orientation value words, such as *patriotic* and *contribution*. It can be deduced that contemporary Chinese values have two orientations. Moreover, this survey also collected *patriotic*, *freedom*, *honesty*, *kind-hearted* and other core socialist values words. This partially reflected the internalization of the core socialist values by contemporary Chinese people.

## 3. Study 2: a text analysis of Chinese values

The open-ended survey in Study 1 revealed that the variety and frequency of socially oriented value words were lower than those of individual-oriented values, which on the one hand, demonstrated that contemporary Chinese values are predominantly individual-oriented. On the other hand, the survey approach likely made individuals prioritize thinking about individual orientation values that are more closely related to them. Since entering a new era, China has placed greater emphasis on promoting the Chinese spirit and Chinese values. Indeed, China’s concept of development, new security concept, global governance concept, etc., all reflect contemporary Chinese values. At the same time, it has strengthened the construction of the core socialist value system in China and actively cultivated and practiced core socialist values. Study 1 focused on the values of the Chinese public. Contemporary Chinese include not only the public but also the Party and state leaders who lead the direction of national development. Therefore, exploring the values of contemporary Chinese people should not only stop at the public’s perception of values but also focus on the expression of the country’s values. Therefore, compiling a list of values words appropriate for Chinese people also needs to take into account current Chinese social values. Based on this, a textual analysis was performed to collect contemporary Chinese social value vocabulary to further enhance the contemporary Chinese value glossary.

### 3.1. Materials and methods

#### 3.1.1. Materials

Xi Jinping Thought on Socialism with Chinese Characteristics for a New Era is the essence of the times of Chinese culture and the Chinese spirit. *Xi Jinping: The governance of China (Volumes I–IV)*, which profoundly reflected Socialist Thought with Chinese Characteristics for a New Era, contained a rich expression of contemporary Chinese values as well as the transmission of traditional values. The books profoundly reflected the social values of contemporary Chinese people. Therefore, this study identified *Xi Jinping: The Governance of China (Volumes I–IV)* as the source of analysis.

#### 3.1.2. Procedure

The researcher read the full text of *Xi Jinping: The Governance of China (Volumes I–IV)* word by word, excerpted independent statements and passages involving expressions of Chinese values, and subsequently built a corpus of value phrases. Even if a value was mentioned several times in the book, it is recorded only once. For example, if a core socialist value was identified in all four books, it was recorded only the first time it appeared. The main contents included: the spirit and traditional virtues of the Chinese nation, the various spirits formed during the development of the country (e.g., the great anti-epidemic spirit, the spirit of the Winter Olympics), and the development concepts promoted by the country (e.g., green values, global governance concepts, common values for all mankind).

### 3.2. Results and discussion

In total, 63 articles in the book referred to values, and a total of 417 value words (phrases) were extracted. The 417 value words were saved in the txt format, and preliminary word frequency statistics were conducted using BICOMB 2.0 software. Finally, words with similar meanings were grouped and combined to obtain a frequency distribution of 56 value words (see Table 2).

**Table 2:** Words and their frequencies obtained by text analysis.

Values	Frequency	Values	Frequency
和谐(harmony)	22	科学(respect for sciences)	5
奋斗(strive)	20	互鉴(mutual learning)	5
包容(inclusiveness)	20	理想(ideal)	4
诚信(honesty)	19	坚强(forcefulness)	4
发展(develop)	18	富强(prosperity)	4
友善(kind-hearted)	16	负责(responsibility)	4
创新(innovate)	15	独立(independent)	4
正义(justice)	14	自省(introspection)	3
勤俭(hardworking and thrifty)	14	自律(self-discipline)	3
民本(people-oriented)	13	坦荡(frank)	3
和平(peace)	13	名节(reputation)	3
共赢(win-win)	13	民主(democracy)	3
开放(openness)	12	廉洁(clean)	3
互利(mutual benefit)	12	敬业(dedication)	3
忠诚(loyalty)	11	健康(health)	3
团结(unity)	11	奉献(contribution)	3
环保(environmental)	11	自由(freedom)	2
剬平(fairness)	11	知耻(shame-awareness)	2
爱国(patriotic)	11	真理(seeking truth)	2
平等(equality)	10	文明(civilization)	2
合作(cooperation)	9	踏实(earnest)	2
大同(Datong)	8	谦虚(modesty)	2
亲情(family affection)	7	谨慎(cautious)	2
求是(be practical and realistic)	6	互信(mutual trust)	2
互助(mutual help)	6	共商(co-discussing)	2
法治(rule of law)	6	共建(co-building)	2
尊重(respect)	5	自信(self-confidence)	1
勇敢(brave)	5	感恩(gratitude)	1

As presented in Table 2, the top five items that the participants valued most were *harmony, strive, inclusiveness, honesty,* and *develop*. Harmony was at the top of the list. Since ancient times, the Chinese have had a deep-rooted desire to pursue *harmony* (Huang, 2016). Besides, previous empirical studies have demonstrated that contemporary Chinese people still attach enormous importance to the value of harmony (Yu et al., 2016; Zhong et al., 2022). On the one hand, Table 2 delineates that contemporary Chinese social values include many traditional values such as *harmony*, *honesty*, *kind-hearted*, and *Datong*, which reflect contemporary Chinese inheritance and the development of traditional Chinese culture. On the other hand, the social values of the Chinese also contained some value words that reflected the characteristics of the times, such as *strive*, *develop,* and *innovate*, which suggested that the values of contemporary Chinese were characterized by modernity. In general, Chinese social values express the characteristics of both tradition and modernity.

The value vocabulary gathered from Study 1 revealed the value characteristics of the general public, while the vocabulary obtained from the text analysis in this study reflected the social value pursuits of contemporary Chinese. The words in the two glossaries were then individually compared. The comparison demonstrated that the public’s existing values (already) and the socially advocated values of the Chinese (should be) overwhelmingly overlapped. Contemporary Chinese values exhibit a socio-cultural dependency. At the same time, there were differences between the two types of values; unlike socially advocated values, public values also included some individual-oriented value pursuits (e.g., *love*, *friendship*, *money*, *happiness*, *power*, and *status*). Likewise, there were some values in socially advocated values that were lacking in the current public values (e.g., *mutual benefit*, *win-win*, *clean*, etc.). The results of this study reflected the values that contemporary Chinese should have, while the results of Study 1 reflected the values that contemporary Chinese actually have. The Chinese have a disconnect between what should be and what is, and there is a discrepancy between the values advocated by society and their own preferred values (Yang, 1998). The socially advocated values obtained in Study 2 were more values that fall under the category of collectivism, but do not mean that the government ignored the development of the individual, instead it integrated the development of the individual into the development of the society. Therefore, some values that lean toward individualism (e.g., *money*, *romantic love*) were not purposely emphasized. Some of the socially advocated values in Study 2 did not appear in the results of Study 1, possibly due to the lack of internalization of these values. Since the core socialist values have been strongly promoted in the past 10 years, especially the values of *patriotic* and *honest* at the individual level were highly internalized and thus had a high frequency in Study 1. The values such as *win-win* and *mutual benefit*, have been promoted for a shorter period of time and thus did not appear or appear with low frequency in Study 1. In addition, values are closely related to the self. Values with a high level of importance are more closely linked to the self (Yue et al., 2022). Although the Chinese self contains multiple selves, some studies have revealed that the individual self is more dominant (Wang P. et al., 2022). Thus, in the open-ended survey of Study 1, individuals were more likely to report personally oriented values and did not give priority to thinking of socially advocated values. This demonstrated that public values may lag behind socially advocated values by one step.

## 4. Study 3: preliminary application of the Chinese value word list

In Study 1, the public values of the Chinese were initially explored through an open-ended survey. In Study 2, the socially advocated values of the Chinese since the new era were explored through textual analysis. Consequently, the two studies reflected the characteristics of contemporary Chinese values from different perspectives. In order to gain a deeper understanding of the value characteristics of contemporary Chinese, the value words identified from the above two studies were further integrated and combined with the value vocabulary from existing studies to form a word list of contemporary Chinese values. Lastly, a preliminary application of the word list was made to explore the value characteristics of contemporary Chinese.

### 4.1. Materials and methods

#### 4.1.1. Participants

Two thousand Chinese participated in this survey, and 1,814 valid data from 645 men and 1,169 women were recovered. The age distribution ranged from 15 to 63 years old (*M* = 24.33, *SD* = 4.18). The participants came from all walks of life, including students, teachers, business managers, civil servants, and so on. In terms of educational background, secondary education or below, tertiary education, and master’s degree and above accounted for 5.13%, 80.37%, and 14.50% of all participants, respectively. Participants in the survey came from 30 provinces on the Chinese mainland.

#### 4.1.2. Procedure

The material used was a self-compiled word list of contemporary Chinese values which contained 76 value words. The word list formation process consists of the following steps. In the first step, vocabulary from previous studies was collected. (1) The vocabulary involved in the classic foreign values measurement instruments (e.g., SVS, RVS). (2) The vocabulary involved in Chinese values measurement instruments (e.g., CVQ, CVS). (3) The vocabulary mentioned in other Chinese disciplines in their research on values. A total of 555 values vocabularies were collected in this step. In the second step, nine psychology students and 13 non-psychology students rated the values vocabulary. Words with the same meaning were merged, words with significantly different meanings were split, and words that were rare, difficult to understand, or ambiguous were deleted and replaced. 160 words were retained in this step. In the third step, the words from Study 1 and Study 2, as well as the words collected in the literature, were combined and the words were rated by 13 students (the rating method was the same as in the second step). This step retained 139 words. In the fourth step, words were deleted and identified. Firstly, 73 participants rated the importance of each word (rating range of 0–9) and deleted words that were not rated highly (words with mean score below 6.3). This method was revealed to delete fewer words, so a second method was used in which 87 participants selected the 5 value words they considered most important from each of the 139 value words and deleted words with low frequency (words with 5 frequency words). Finally, 76 valid words were retained.

The study procedure was identical to that of Study 1, and the participants filled out the questionnaire by clicking on the link or scanning the QR code. During the survey, participants were asked to select the 5 value words that they valued most. Each participant was given 2 RMB as compensation.

### 4.2. Results and discussion

The five value words selected by the participants were first analyzed. The co-word analysis method was used based on a previous study (Guo et al., 2015). The co-word analysis method is a type of content analysis approach where a higher frequency of word co-occurrence indicates a closer connection between words (Zhong and Li, 2008). Thus, the relationship between value words can be determined based on the frequency of the co-occurrence of words.

#### 4.2.1. Frequency analysis of the value words

The data were organized, the words were saved in the txt format, and the BICOMB 2.0 software was used for word frequency statistics (Table 3).

**Table 3:** Frequency distribution of 76 value words.

Values	Frequency	Values	Frequency
健康(health)	630	聪明(intelligent)	86
爱国(patriotic)	406	认真(seriously)	85
诚信(honesty)	371	机遇(opportunity)	82
亲情(family affection)	349	踏实(earnest)	81
自由(freedom)	290	自省(introspection)	75
自信(self-confidence)	275	谦虚(modesty)	75
快乐(happiness)	273	友情(friendship)	73
乐观(optimism)	257	陪伴(accompany)	72
尊重(respect)	243	远见(forward-looking)	70
感恩(gratitude)	224	勤俭(hardworking and thrifty)	66
金钱(money)	203	坦荡(frank)	63
自律(self-discipline)	202	共赢(win-win)	62
平等(equality)	195	互助(mutual help)	61
独立(independent)	194	合作(cooperation)	59
能力(capacity)	181	地位(status)	56
剬平(fairness)	177	真理(seeking truth)	55
友善(kind-hearted)	172	廉洁(clean)	54
正义(justice)	161	发展(develop)	52
奋斗(strive)	158	互信(mutual trust)	50
忠诚(loyalty)	146	科学(respect for sciences)	47
法治(rule of law)	146	知耻(shame-awareness)	45
勇敢(brave)	141	容貌(appearance)	44
坚强(forcefulness)	140	环保(environmental)	40
负责(responsibility)	136	互利(mutual benefit)	38
民主(democracy)	134	荣誉(glory)	37
爱情(romantic love)	131	人情(ren qing)	35
理想(ideal)	131	求是(be practical and realistic)	31
敬业(dedication)	130	渊博(erudite)	30
和平(peace)	124	谨慎(cautious)	30
文明(civilization)	123	认可(be recognized by others)	28
富强(prosperity)	122	开放(openness)	26
团结(unity)	120	慷慨(generous)	20
创新(innovate)	117	享乐(hedonist)	15
包容(inclusiveness)	114	名节(reputation)	15
和谐(harmony)	107	竞争(compete)	11
信仰(belief)	90	共建(co-building)	9
奉献(contribution)	88	共商(co-discussing)	6
权力(power)	88	互鉴(mutual learning)	3

As illustrated in Table 3, the five values most valued by contemporary Chinese were *health*, *patriotism*, *honesty*, *family affection*, and *freedom*. These five values were also found to be highly ranked in Study 1, which validated the consistency of the results obtained from the different research methods. At the same time, the ranking of some values was altered in this study compared to Study 1; for example, the importance of *patriotic* values moved from 10th to 2nd place, indicating a stronger social responsibility among contemporary Chinese. Similarly, some studies have discovered the national social component contained in Chinese values (Xin and Jin, 2006; Yu et al., 2016). At the same time, it also reflected the inheritance of the traditional Chinese values of family and country. Interestingly, the importance of the value of *honesty* moved up from the 8th rank to the third place. Honesty reflects morality in Chinese values. The core values of Confucian ethics are the “Five Constants” (五常), i.e., 仁 (benevolence), 义 (righteousness), 礼 (propriety), 智 (wisdom), and 信 (honesty). Hence, *Honesty* is one of the “Five Constants” and is a traditional Chinese core value. According to various studies, integrity values are an important element of contemporary Chinese values (Wang and Sun, 2017; Ye et al., 2018), and the finding that integrity values were ranked high in this study are in line with the observations of previous studies. Additionally, the importance of the value of *freedom* rose from 7th to 5th place. The importance of *freedom* in contemporary Chinese was influenced by Western liberal beliefs on the one hand and reflected the inheritance of the traditional Chinese Taoist concept on the other hand. Both Taoism and Confucianism are the philosophical roots of Chinese cultural values (Song, 2012). The former is concerned with physical training and cultivation, promoting less desire and focusing on inner peace and spontaneity (Weng et al., 2021). *Patriotism*, *honesty,* and *freedom* are all components of socialist core values, and the high ranking of these three values partly reflected the internalization of socialist core values. In addition, values within personal emotion, such as *friendship* and *romantic love,* were less important compared to Study 1. Collectively, participants prioritized values that were closely related to their personal development in the open-ended survey, while the importance of some socially advocated values was highlighted when a forced selection method was applied.

#### 4.2.2. Characteristics of contemporary Chinese values on demographic variables

A score of 1 was assigned to the values selected by the participants, while values that were not selected were assigned a score of 0. The total score of each value in different groups was calculated to obtain the results of ranking Chinese people’s values under different demographic variables (see Supplementary material). On the gender variable, the overall ranking of values for males and females was significantly positively correlated, *r* = 0.95, *p* < 0.01. This indicated that overall the ranking of values for participants of different genders was relatively similar. Further examining whether there were differences in the scores of participants by gender on specific values, the Mann–Whitney *U*-test showed significant gender differences in the values of *science*, *democracy*, *romantic love*, *power*, *optimism*, *appearance*, and *respect*. Specifically, males placed more weight on *science* (Z = 3.79, *p* < 0.001), *democracy* (Z = 3.25, *p* < 0.01), *romantic love* (Z = 4.06, *p* < 0.001), and *power* (Z = 2.22, *p* < 0.05), and females emphasized *optimism* (Z = 2.16, *p* < 0.05), *appearance* (Z = 2.12, *p* < 0.05), and *respect* (Z = 2.22, *p* < 0.05) more than males. On the age variable, the overall ranking of values was significantly positively correlated across participants at different ages, with a correlation coefficient *r* ranging from 0.79 to 0.95, *p* < 0.01. This indicated that the ranking of values was relatively consistent across participants at different ages. The Kruskal-Wallis test was used to examine the differences in specific values among participants of different age groups and found significant age differences in the values of *environmental*, *dedication*, *hardworking and thrifty*, *health*, *money*, *happiness*, *accompany*, *appearance*, and *freedom*. Specifically, participants in the 31–40 age group placed more importance on *dedication* (Z = 3.41, *p* < 0.01), and *money* (Z = 2.76, *p* = 0.059) than those under 25 years of age. And participants under 25 years of age placed more importance on *happiness* (Z = 3.29, *p* < 0.05), and *appearance* (Z = 3.04, *p* < 0.05) than participants aged 31–40 years. Participants in the 41–50 age group gave more importance to *environmental* (Z = 3.59, *p* < 0.01) and *hardworking and thrifty* (Z = 3.32, *p* < 0.01) than the 25 age group. And participants in the 41–50 age group viewed *hardworking and thrifty* more than the 26–30 age group (Z = 3.08, *p* < 0.05). Participants over 50 years old placed more importance on *accompany* than those under 25 years old (Z = 5.25, *p* < 0.001), 26–30 years old (Z = 5.68, *p* < 0.001), 31–40 years old (Z = 5.35, *p* < 0.001), and 41–50 years old (Z = 4.03, *p* < 0.001), and were more *health* oriented than those under 25 years old (Z = 2.76, *p* = 0.059). In addition, participants under 25 years of age emphasized *freedom* more than those aged 26–30 years (Z = 3.48, *p* < 0.01), 31–40 years (Z = 4.60, *p* < 0.001), and 50 years and older (Z = 3.21, *p* < 0.05). The results of the correlation analysis on the overall ranking of the values of the participants in different regions showed that the ranking of the values in each region was significantly positively correlated, with the correlation coefficient *r* ranging from 0.81 to 0.96, *p* < 0.01. To further compare whether there were significant differences in the scores of the participants in different regions on specific values, the Kruskal-Wallis test revealed that there were significant regional differences in the values of *innovate*, *contribution*, and *prosperity*. Specifically, *innovate* (Z = 3.20, *p* < 0.01) and *prosperity* (Z = 2.63, *p* = 0.052) were more important in the western region than in the northeastern region, and *contribution* was more important in the northeastern region than in the eastern region (Z = 3.43, *p* < 0.01), central region (Z = 3.35, *p* < 0.01), and western region (Z = 3.65, *p* < 0.01).

#### 4.2.3. High-frequency word analysis

**Figure 1:**
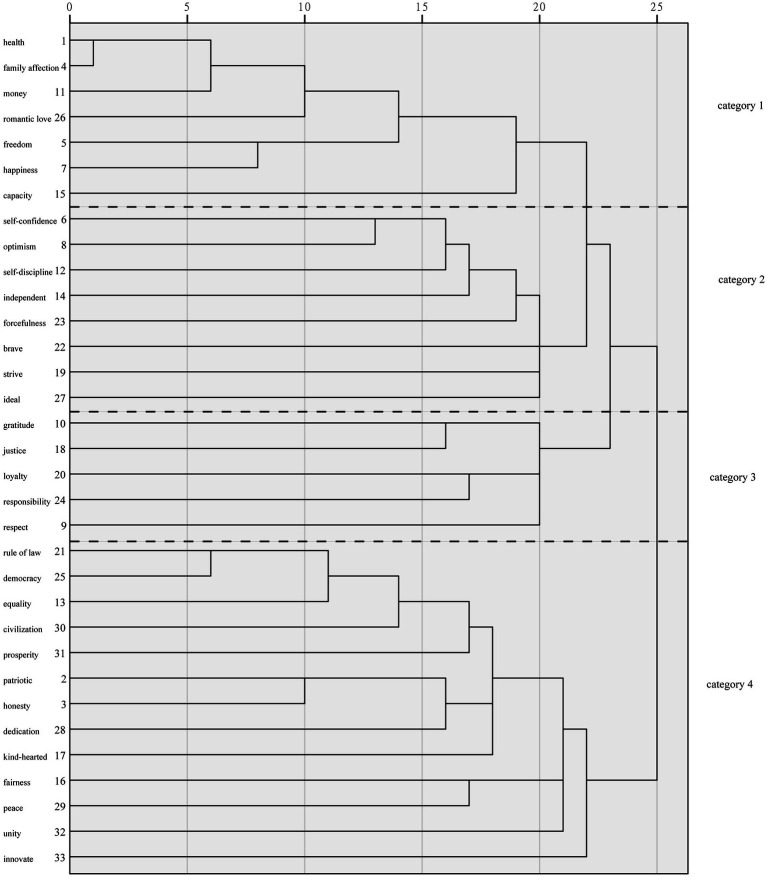
Results of clustering analysis of high-frequency value words.

As shown in Table 3, the cumulative percentage of word frequency of the first 33 value words exceeded 75%; therefore, the first 33 words were used as high-frequency words for the ensuing analysis. To begin, the 33 high-frequency words were intercepted in BICOMB 2.0 software for co-occurrence analysis to generate a word-part matrix, which was subsequently imported into SPSS 24.0 for cluster analysis (Figure 1). The Ochiia coefficient, which denotes the probability of co-occurrence of two keywords and lies between 0 and 1, was selected to generate a similarity matrix of high-frequency words. Next, the similarity matrix was transformed into a dissimilarity matrix. The dissimilarity matrix reflected the similarity between two words; the closer the value is to 0, the greater the similarity between the two words and the closer the correlation; consequently, the closer the value is to 1, the further the distance between the two words and the lower the similarity (Guo, 2016).

As displayed in Figure 1, contemporary Chinese values can be roughly divided into four categories. The first category of values encompassed the goals and pursuits that individuals want to achieve in materialistic, spiritual, physical, and psychological terms, commonly referred to as *self-fulfillment*. The second category of values was chiefly associated with essential qualities that individuals possessed in the pursuit of their own development, the methods and means that individuals needed to achieve their goals, and was named *self-cultivation*. The third category of values described the positive qualities that individuals held in their interactions with others to achieve harmonious interpersonal development and was termed *interpersonal ethics*. The last category of values was primarily concerned with the social values in the individual value system, named *social development*.

**Figure 2:**
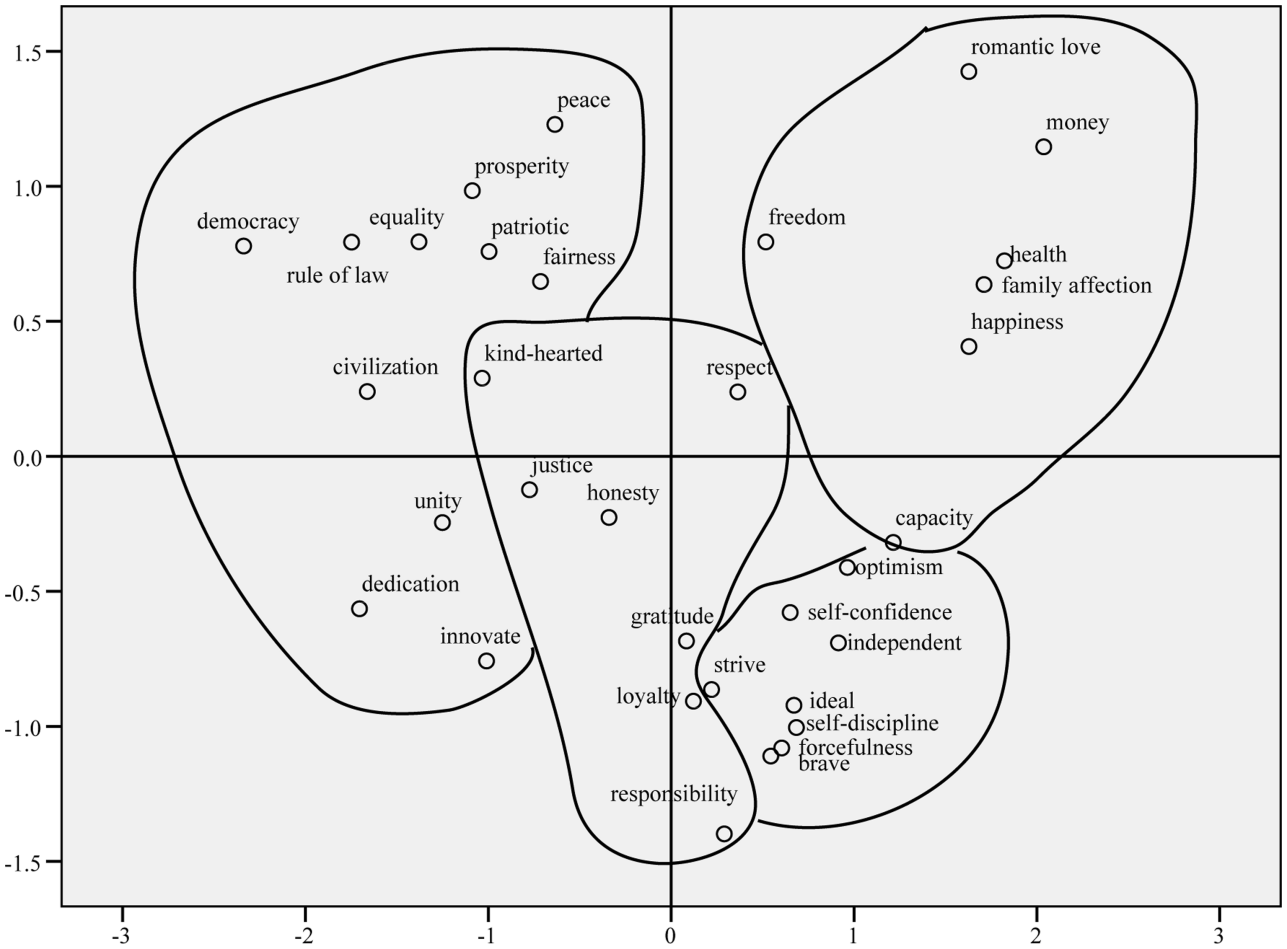
Multidimensional scale results of high-frequency value words.

Multidimensional scale analysis (MDS) was performed on the high-frequency word dissimilarity matrix in order to clearly display the relationship between the value words and to generate their two-dimensional spatial distribution map. The two-dimensional spatial distribution map was divided into regions and marked with lines according to the spectral map of cluster analysis (see Figure 2).

As delineated in Figure 2, there were two dimensions in the spatial distribution of contemporary Chinese values. The horizontal axis represented the social-individual dimension, with the left part being socially oriented values and the right part being individually oriented values. The vertical axis represented the instrumental-goal dimension, with the bottom part being the means values and the top part being the goal values. After inserting the four categories of values from the cluster analysis into a two-dimensional spatial distribution, *self-fulfillment* was basically located in the “individual-goal” region, and *self-cultivation* was exclusively located in the “individual-instrumental” region. *Social development* belonged entirely to the “social” region, and *interpersonal ethics* was located in the middle of the four regions.

## 5. General discussion

### 5.1. The diversity characteristics of contemporary Chinese values

First, from the perspective of value orientations, Chinese values possessed both individual and social orientations, in agreement with the observations of prior studies (Jin et al., 2009; Yu et al., 2016). In this research, the open-ended survey in Study 1 collected both orientations of value words, and Study 3 further delineated individual and social orientations through multidimensional scale analysis, both of which corroborated that contemporary Chinese values had individual and social orientations. Secondly, in terms of the nature of values, Chinese values include instrumental values and goal values. Rokeach (1973) classified values into instrumental values and terminal values. Meanwhile, other researchers have also distinguished between instrumental and goal values in their exploration of Chinese values (Hsu and Huang, 2016; Li and Huang, 2016). The multidimensional scale analysis in this study also confirmed that both instrumental values and goal values coexist in the value system of contemporary Chinese. Furthermore, Chinese values are characterized by the co-existence of tradition and modernity. Traditional Chinese culture, especially Confucianism, emphasized the pursuit of morality and virtue, so traditional Chinese values featured a strong moral character. Herein, the *interpersonal ethics* dimension reflected the transmission of traditional Confucian values to contemporary Chinese people. The modernization of China into cities has not led to a complete shift in Chinese values, and traditional Chinese values are still deeply rooted (Herdin and Aschauer, 2013). This study also found that modern values such as *democracy* and *equality* also played a significant role in the value system of contemporary Chinese. It can be observed that contemporary Chinese values were characterized by the co-existence of tradition and modernity, and this result was consistent with the findings of previous studies (Faure and Fang, 2008; Hsu and Huang, 2016).

### 5.2. Validity of research methods

The most common research methods used in previous value studies were the rating method and the ranking method. Nevertheless, these scoring methods suffer from certain shortcomings. Since value items all have positive attributes, it is challenging to make large distinctions in assessing the importance of values, and participants tend to score items highly, making it difficult to conclude which values are truly important to individuals (McCarty and Shrum, 2000). Besides, when the ranking method is used, individuals may not be able to reliably distinguish the importance of different items when there are many valuable items, which can lead to inaccurate results (Harzing et al., 2009). The selection method can only yield a few values that individuals value most, but the relative order of values cannot be identified for each participant. Hence, each value research approach has specific advantages and limitations. In Study 3, the word choice method was applied, and the results were consistent with those of previous studies, indicating the feasibility of this method for value research. In addition to using the word choice method, an open-ended survey of values was also implemented in Study 1. The relative consistency of the top-ranked value words of the participants in the two studies substantiated the validity of the research method used in this study.

### 5.3. Internalization of social values and its educational revelation

In recent years, the Chinese government has promoted several social values, especially the core socialist values. So have these social values been internalized into the personal value system of contemporary Chinese? In both Study 1 and Study 3, it was found that some social values (e.g., *patriotism* and *honesty*) appeared with high frequency, insinuating that these social values have become critical values in the value system of contemporary Chinese. More importantly, the cluster analysis of Study 3 found that contemporary Chinese values can be divided into four categories, among which the *social development* dimension contained value words that belong to the socially advocated values, and the majority of them were core socialist values. Moreover, this study exposed that with the popularization and education of core socialist values, these values are becoming more and more deeply rooted and gradually internalized into the values of contemporary Chinese. At the same time, it should be noted that the degree of internalization of some social values may not be sufficient. In the open survey, some words of core socialist values were mentioned at a low frequency (e.g., *dedication* and *prosperity*), which implied that individuals are more inclined to take their own set of values as guidance in daily life. However, in study 3, words of core socialist values were frequently selected, meaning that despite the core values being positively recognized cognitively, some core values may not be included in the self-concept. Strikingly, some social value words mentioned in Study 2, especially those that have been widely mentioned in recent years (e.g., *co-building* and *co-discussing*), were not actively mentioned by the participants in Study 1, and the frequency of these words in Study 3 was also very low. Why are some social values highly internalized while others are less internalized? On the one hand, it has to do with the duration of time that social values have been promoted. It has been more than 10 years since the core socialist values were proposed, and the slogans that can be seen everywhere and the emphasis on school education have made it possible for the core socialist values to gradually penetrate into the hearts of contemporary Chinese people. Therefore, in order to realize the internalization of social values, values education still needs to be strengthened. On the other hand, the extent to which values are strongly linked to the self may influence the internalization of social values. It has been shown that values of high importance levels are closely linked to the self (Brosch et al., 2012; Yue et al., 2022). In this study, values such as *honest* and *patriotic* belonged to the individual level of core socialist values, which were more closely connected to the individual’ self and appeared more frequently compared to values such as *prosperity* and *democracy* at the national level of core socialist values. Therefore, strengthening the connection between social values and the self may be a direction for values cultivation in the future.

### 5.4. Limitations and future research

There are still many shortcomings in this study that need to be considered. This study compiled a vocabulary of contemporary Chinese values and made a preliminary application of the vocabulary to explore the characteristics of Chinese values. In Study 3, only the selection method was used. As previously mentioned, each method has certain limitations. In subsequent studies, rating, ranking, and other approaches should be combined to validate the results of this study. This value vocabulary could only examine the explicit value characteristics of contemporary Chinese people. The experimental paradigm of implicit measurement should also be utilized in social cognition and projection methods to further investigate the implicit value of Chinese individuals. In addition, with the development of The Times, people’s need for a better life is growing, and the values of the Chinese will change accordingly. In the future, words in the word list need to be supplemented.

## 6. Conclusion

This study collected values words through an open-ended survey and a textual analysis, on the basis of which a list of contemporary Chinese values words was compiled and used to explore the values of contemporary Chinese people. The findings of this study verified the value structure theory proposed by Yang (1998) and also confirmed that contemporary Chinese values are characterized by the coexistence of tradition and modernity, which initially revealed the value characteristics of Chinese people in the new era. At the same time, the value words obtained in this study basically contain the values that contemporary Chinese people look for, and can be used to examine the values of Chinese people in the new era, and also to develop a Chinese values questionnaire based on them. In addition, it was found in the study that among the socially advocated values, those that have been advocated for a longer period of time or are more closely associated with individuals are more internalized, which can provide insight into the internalization of social values.

## Data availability statement

The raw data supporting the conclusions of this article will be made available by the authors, without undue reservation.

## Ethics statement

The studies involving human participants were reviewed and approved by the Faculty of Psychology, Southwest University. The patients/participants provided their written informed consent to participate in this study.

## Author contributions

LP: conceptualization, methodology, formal analysis, investigation, writing—original draft, writing—review, editing, and visualization. XH: conceptualization, writing—review, editing, supervision, project administration, and funding acquisition. All authors contributed to the article and approved the submitted version.

## Funding

This study was supported by a major project of the National Social Science Foundation, the theoretical construction and practical path research of Chinese community Psychology in the New Era (22&ZD184) and Chongqing Humanities and Social Sciences Key Research Base Project “Study on the Competence Characteristics of Community Leaders” (18SKB003).

## Conflict of interest

The authors declare that the research was conducted in the absence of any commercial or financial relationships that could be construed as a potential conflict of interest.

## Publisher’s note

All claims expressed in this article are solely those of the authors and do not necessarily represent those of their affiliated organizations, or those of the publisher, the editors and the reviewers. Any product that may be evaluated in this article, or claim that may be made by its manufacturer, is not guaranteed or endorsed by the publisher.
